# Development and application of loop-mediated isothermal amplification for detecting the highly benzimidazole-resistant isolates in *Sclerotinia sclerotiorum*

**DOI:** 10.1038/srep17278

**Published:** 2015-11-26

**Authors:** Ya Bing Duan, Ying Yang, Jian Xin Wang, Cong Chao Liu, Ling Ling He, Ming Guo Zhou

**Affiliations:** 1 College of Plant Protection, State & Local Joint Engineering Research Center of Green Pesticide Invention and Application, Nanjing Agricultural University, Nanjing, 210095, China

## Abstract

Resistance of benzimidazole fungicides is related to the point mutation of the *β*-tubulin gene in *Sclerotinia sclerotiorum*. The point mutation at codon 198 (GAG → GCG, E198A) occurs in more than 90% of field resistant populations in China. Traditional detection methods of benzimidazole-resistant mutants of *S. sclerotiorum* are time-consuming, tedious and inefficient. To establish a suitable and rapid detection of benzimidazole-resistant mutants of *S. sclerotiorum*, an efficient and simple method with high specificity was developed based on loop-mediated isothermal amplification (LAMP). Eight sets of LAMP primers were designed and four sets were optimized to specially distinguish benzimidazole-resistant mutants of *S. sclerotiorum*. With the optimal LAMP primers, the concentration of LAMP components was optimized and the reaction conditions were set as 60–64 °C for 60 min. This method had a good specificity, sensitivity, stability and repeatability. In the 1491 sclerotia, 614 (41.18%) were positive by LAMP, and 629 (42.19%) positive by MIC. Therefore, the LAMP assay is more feasible to detect benzimidazole-resistant mutants of *S. sclerotiorum* than traditional detection methods.

*Sclerotinia sclerotiorum* (Lib.) de Bary is a most destructive fungal plant pathogen which attacks more than 400 plant species, including many agronomic and horticultural crops^1^,^2^,^3^. Because breeding programs for disease resistance have been hampered by limited gene sources, application of fungicides is the principal tool for controlling Sclerotinia diseases on most crops^4^,^5^,^6^. Unfortunately, the extensive use of a single fungicide will cause the emergence of resistant populations in *S. sclerotiorum*, leading to invalid control. Therefore, monitoring fungicide resistance is a very important work in risk assessment of fungicide resistance and integrated control of Sclerotinia diseases.

Carbendazim (MBC), one typical benzimidazole fungicide, has been extensively used to control this disease routinely since the early 1980s^7^. At present, MBC-resistant populations of *S. sclerotiorum* has appeared and been increasing with the service time. However, MBC was still used to control Sclerotinia diseases in field. In order to control Sclerotinia disease effectively, it is necessary to monitor MBC resistance over a large region. Previous detection methods have been established by mycelial growth inhibition on PDA containing the different concentrations^8^,^9^, but the procedure is time-consuming and tedious with low efficiency. With the development of molecular biology technology, PCR detection techniques were developed in recent years based on molecular resistance mechanism of *S. sclerotiorum* to MBC^10^,^11^. The methods could detect MBC resistance much more rapidly than the classical mycelial growth inhibition. However, they were limited by the requirement of expensive instruments, insufficient specificity, and rather low amplification efficiency. Developing a rapid, economic and high-throughput technique is very necessary for detecting MBC-resistant populations in *S. sclerotiorum*.

Loop-mediated isothermal amplification (LAMP) is an outstanding gene amplification procedure, which can amplify nucleic acids with high specificity, sensitivity, efficiency and rapidity under isothermal conditions^12^. This technique is characterized by the use of a DNA polymerase with strand displacement activity and a set of two specially designed inner primers (FIP and BIP) and two outer primers (F3 and B3)^13^,^14^. LAMP had high specificity for the target sequence because the target sequence was recognized by six independent sequences (F1c, F2, F3, B1c, B2 and B3) in the initial stage and by four independent sequences (F1c, F2, B1c, and B2) in the later stages of the LAMP reaction (Fig. S1). The amplification efficiency of the LAMP method was extremely high based on the isothermal reaction. Therefore, the LAMP assay had advantages in specificity, selectivity and rapidity over other nucleic acid amplification methods^13^. Moreover, Nagamine *et al.* had improved the method by putting forward loop primers (LF and LB) that accelerated the LAMP reaction^15^.

Monitoring fungicide resistance in pathogen populations is a critical stage in designing effective fungicide application strategies. A LAMP assay for the resistant populations of *S. sclerotiorum* to benzimidazole fungicides was developed and demonstrated to be efficient in fields. The new LAMP assay will be used for early warning of resistance risk of *S. sclerotiorum* to benzimidazole fungicides in fields and provide important reference data for control of Sclerotinia diseases caused by *S. sclerotiorum*.

## Results

### Specificity of LAMP primers

**Figure 1:**
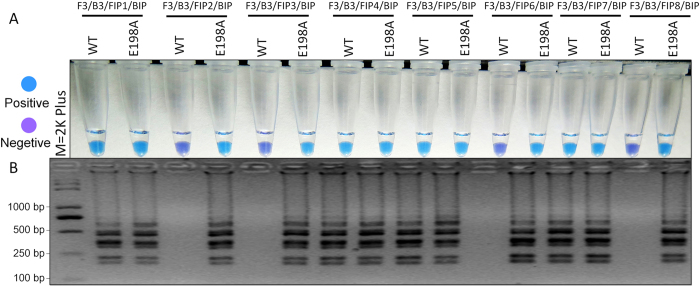
Optimization of LAMP primers. (**A**) Assessment was based on HNB visualization of color change of the LAMP products. (**B**) Assessment was based on gel electrophoresis analysis of the LAMP products.

Optimization of LAMP primers is essential for accurate and specific detection of the LAMP method. To increase the likelihood that the LAMP primers would specifically detect the highly benzimidazole-resistant mutants (E198A), one or two mismatches were added to the 5′ end of FIP and eight sets of LAMP primers (Table S1) were obtained and tested in this study. Based on HNB-visualized color change and the ladder-like pattern on gel electrophoresis, four sets of LAMP primers were appropriate for detecting the highly benzimidazole-resistant mutants of *S. sclerotiorum* (Fig. 1). The positive sample was indicated by a sky blue color; while the negative sample remained violet color (Fig. 1A). After assessment by color change, it was reconfirmed by gel electrophoresis. As expected, the typical ladder-like pattern on 3.0% gel electrophoresis was observed in a positive sample rather than the negative control (Fig. 1B). The results indicated that four primer sets (Fig. 1, Table S1) could be used to specifically distinguish the highly benzimidazole-resistant mutants of *S. sclerotiorum*. The primer set S2 was randomly selected for the further tests in this study.

### Optimization of LAMP reaction components

**Figure 2:**
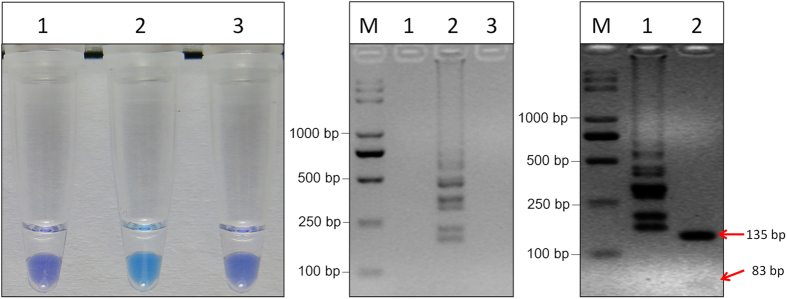
LAMP detection of the highly MBC-resistant mutants in *S. sclerotiorum* and digestion of the positive LAMP products. (**A**) LAMP detection of the highly MBC-resistant mutants with HNB-visualization. The reaction becomes sky blue if the *β-*tubulin gene has a point mutation at codon 198 but remains violet if the *β-*tubulin gene has no mutation or other mutation at codon 198. 1, HA61; 2, TZ25; 3, ddH_2_O. (**B**) Agarose gel electrophoresis of LAMP products. The positive reaction is manifested as a ladder-like pattern on the 3.0% agarose gel. M, 2K plus; 1, HA61; 2, TZ25; 3, ddH_2_O. (**C**) LAMP products were digested with Alu I, and two fragments (135 bp, 83 bp) were observed by 3.0% agarose gel. M, 2K plus; 1, LAMP products without digestion; 2, LAMP products digested by Alu I.

With genomic DNA of the mutant TZ25 and the wild type HA61 as the template, optimization of LAMP reaction components was performed. As expected, visual detection with HNB (Fig. 2A) and the ladder-like pattern on 3% gel electrophoresis (Fig. 2B) were observed in the mutant TZ25 but not in the wild type HA61 and ddH_2_O. Thus, the appropriate reaction was obtained in 10 μL comprising 2.4 U Bst DNA polymerase, 1.0 μL 10×ThermoPol Buffer, 2.0 mM MgCl_2_, 0.8 mM dNTPs, 0.6 M betaine, 100 μM HNB, 1.2 μM each of FIP and BIP, 0.3 μM each of F3 and B3, and 1 ng of target DNA.

### Optimization of LAMP reaction conditions

**Figure 3:**
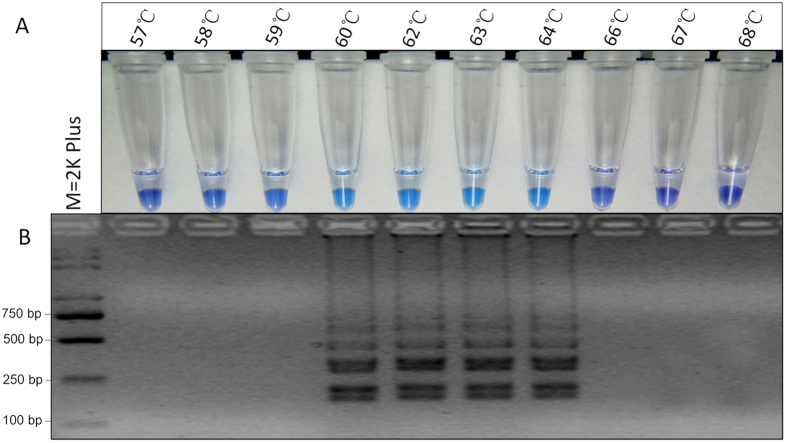
Optimization of LAMP reaction temperature. (**A**) Assessment was based on HNB visualization of color change of the LAMP products. (**B**) Assessment was based on gel electrophoresis analysis of the LAMP products.

**Figure 4:**
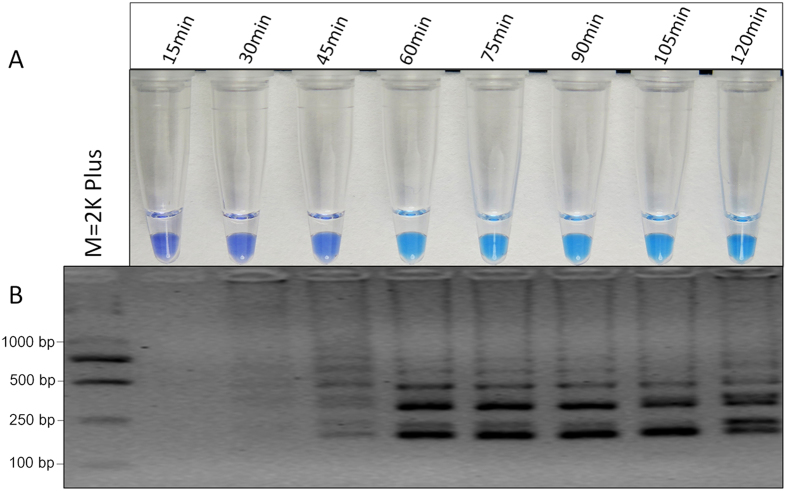
Optimization of LAMP reaction time. (**A**) Assessment was based on HNB visualization of color change of the LAMP products. (**B**) Assessment was based on gel electrophoresis analysis of the LAMP products.

With genomic DNA of the mutant TZ25 as the templates, optimization of reaction temperature and time was performed on basis of the above optimized LAMP reaction components. Color change and the ladder-like pattern of the LAMP products were observed at 60–64 °C (Fig. 3A) and the intensity of DNA was similar (Fig. 3B). With the appropriate temperature (62 °C), the reaction time was optimized and the positive LAMP products were observed from 60 to 120 min by HNB-visualized color change (Fig. 4A) and from 45-120 min by gel electrophoresis (Fig. 4B). When the reaction time reached to 45 min, the color of LAMP products was slightly changed and the intensity of ladder-like pattern on gel electrophoresis was low, indicating the amplification efficiency of LAMP was low at 45 min. Therefore, the appropriate reaction condition of the established LAMP method was set as 60–64 °C for 60 min.

### Validation of the LAMP products

The positive LAMP products was digested with the restriction enzyme Alu I and the 83 and 135 bp bands were observed on agarose gel after electrophoresis (Fig. 2C). The results were in good accordance with those predicted theoretically from the expected structures. To further confirm the LAMP products, the plasmid pET218 was transformed into competent cells and the positive clones were obtained for sequence analysis. The sequencing results showed that the 218 bp target fragment was 100% homologous to the *β-*tubulin gene used for the primers design. These results indicated that the LAMP products were specifically amplified from the *β-*tubulin target region in *S. sclerotiorum*.

### Specificity of LAMP

**Figure 5:**
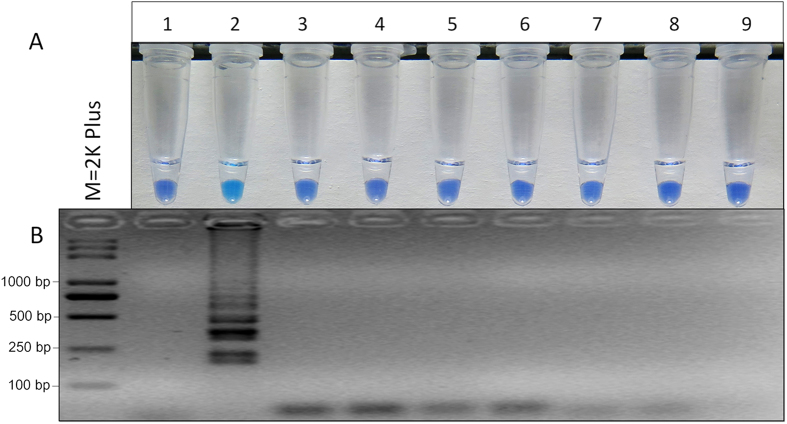
Specificity of LAMP detection of the E198A mutant in *S. sclerotiorum*. Assessment was based on (**A**) HNB visualization of color change or (**B**) gel electrophoresis analysis of the LAMP products. M, 2K plus; 1, wild type of *S. sclerotiorum*; 2, E198A genotype of *S. sclerotiorum*; 3, F200Y genotype of *S. sclerotiorum*; 4, E198K genotype of *F. graminearum*; 5, E198L genotype of *F. graminearum*; 6, E198V genotype of *B. cinerea*; 7, E198A genotype of *B. cinerea*; 8, E198K genotype of *B. cinerea*; 9, ddH_2_O.

The F200Y mutation genotype of *S. sclerotiorum*, the E198K, E198L mutation genotypes of *F. graminearum*, and the E198V, E198A, E198K mutation genotypes of *B. cinerea* were used as negative controls to determine the specificity of LAMP. From HNB-visualization (Fig. 5A) or gel electrophoresis (Fig. 5B), LAMP was positive only for the E198A mutant TZ25 of *S. sclerotiorum*, but not for other mutants or isolates. This indicated that the established LAMP method had a high level of specificity for detecting the E198A mutation genotype of *S. sclerotiorum*.

### Sensitivity of LAMP and PCR

**Figure 6:**
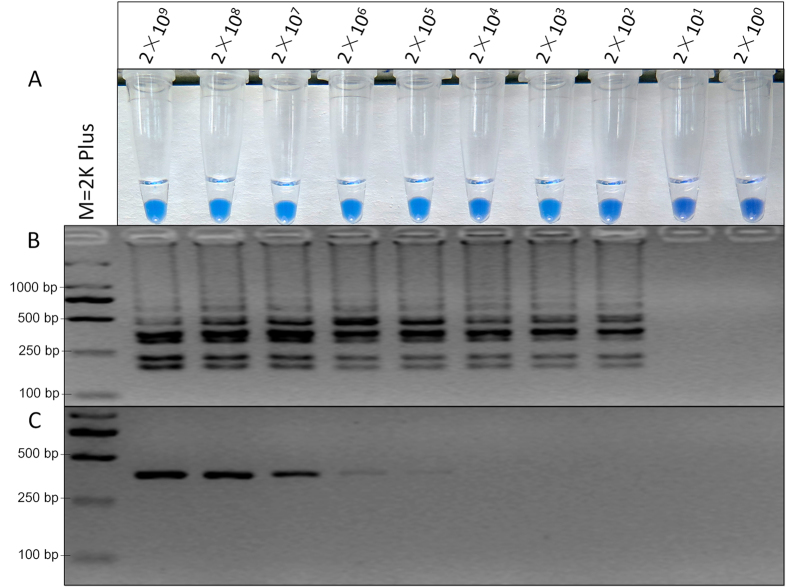
Sensitivity of LAMP vs. conventional PCR. Detection by (**A**) LAMP and HNB visualization, (**B**) LAMP and gel electrophoresis, and (**C**) conventional PCR and gel electrophoresis.

To determine the detection limit, PCR and LAMP were performed with tenfold serial dilutions of the plasmid pET383 as templates. PCR products were detected by gel electrophoresis and LAMP products were detected by HNB-visualization and gel electrophoresis. A 383 bp band was specifically amplified and successfully detected by PCR when the template was diluted to 2 × 10^5^ copies (Fig. 6C). Color change with HNB (Fig. 6A) and the ladder-like pattern (Fig. 6B) of the LAMP products were observed from 2 × 10^9^ to 2 × 10^2^ copies and the detection limit was 2 × 10^2^ copies. Thus, LAMP was 1000-fold more sensitive than PCR.

### Repeatability of LAMP

**Figure 7:**
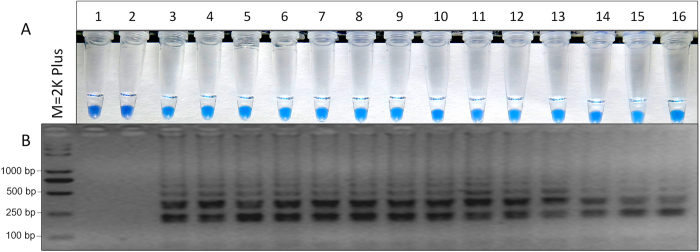
Repeatability of LAMP detection of the E198A mutants in *S. sclerotiorum*. Assessment was based on (**A**) HNB visualization of color change or (**B**) gel electrophoresis analysis of the LAMP products. 1, ddH_2_O; 2, the wild type HA61; 3–16, the known E198A mutants.

Prior to the repeatability test, 14 known E198A mutants from different geographical regions in China (Table S2) were confirmed by MIC and sequence analysis. According to HNB-visualization (Fig. 7A) and gel electrophoresis (Fig. 7B), all the E198A mutants tested for repeatability were positive, but not the wild type and the control ddH_2_O. This indicated that the established LAMP had good robustness and repeatability.

### Comparison of LAMP, PCR and MIC

A total of 864 sclerotia were washed with sterile water and divided into two parts. One part was used for DNA extraction as templates of LAMP and PCR. The other part was tested using the MIC method. The positive-sample ratios were 19.68%, 18.52%, and 20.14% by LAMP, PCR, and MIC, respectively (Table 1). Compared with PCR, resistance frequency from LAMP was more close to that from MIC. In addition, the positive samples detected by LAMP were confirmed by sequence analysis, which showed a good agreement with MIC. This indicated that LAMP established in this study could rapidly monitor the highly MBC-resistance populations of *S. sclerotiorum*.

### Application of LAMP on monitoring MBC-resistance of *S. sclerotiorum* in field

In 2014, 1491 sclerotia from diseased rape stems were tested by MIC and the ratio of the resistant mutants was 42.19% (629/1491) (Table 2). To further verify the application of LAMP on resistance monitoring of *S. sclerotiorum* to MBC in field, LAMP was also performed using DNA of these isolates as templates and the positive-sample ratio was 41.18% (614/1491) (Table 2). The results measured by LAMP were similar to those measured by MIC, indicating that LAMP was very feasible to detect the resistant mutants of *S. sclerotiorum*. Therefore, LAMP reported here might be used for monitoring MBC-resistance populations of *S. sclerotiorum* in field.

## Discussion

Since LAMP was reported in 2000 by Notomi, it has been widely applied in nucleic acid analysis because of its simplicity, rapidity, high efficiency, and outstanding specificity^12^. In recent years, this method has been successfully applied in molecular detection and diagnostics of bacterial^16^,^17^, viral^18^,^19^, fungal^20^,^21^,^22^,^23^, and parasitic diseases^24^,^25^ in both animals and plants. However, the application of LAMP in fungicide resistance of plant pathogens has not been reported until the detection of benzimidazole-resistant mutants of *F. graminearum* was disclosed by our group^26^. In this study, we developed and assessed the simple and rapid LAMP for the detection of the highly benzimidazole-resistant isolates of *S. sclerotiorum*. To our knowledge, it is the first report that LAMP has been applied in detecting fungicide resistance of *S. sclerotiorum*.

One of the advantages of LAMP is its visualization with the naked eyes. The LAMP products can be judged by adding DNA intercalating dyes or metal-ion indicators such as SYBR green^27^, Picogreen^28^, hydroxynaphthol blue (HNB)^29^, CuSO_4_^30^, or calcein^31^. DNA intercalating dyes were added after completion of the reaction. To avoid contamination caused by exposed operation, a visualization indicator (HNB) prior to amplification is used in this study. For HNB visual detection, a positive reaction is indicated by a color change from violet to sky blue, and the negative reactions retains unchanged. Results of HNB-color change were reconfirmed using 3% agarose gel electrophoresis and stained with ethidium bromide. In this study, we established a LAMP assay to detect the highly benzimidazole-resistant isolates of *S. sclerotiorum*, and the amplification result can be observed by HNB-visualized color change using the naked eyes. Compared with the conventional PCR, the developed LAMP does not require a thermal cycler and very suitable for fungicide resistance detection not only in the laboratory but also in the field.

Based on the point mutation (GAG → GCG, E198A) of the *β*-tubulin gene in the benzimidazole-resistant isolates of *S. sclerotiorum*, LAMP primers were designed using the online software Primer explorer V4 program. To increase possibility that LAMP primers could specifically distinguish the E198A mutants from the wild types, one or two mismatches were added to the 5′ end of FIP (Table S1). Eight sets of LAMP primers were used for the optimization and four primer sets were unexpectedly obtained. In the previous study^26^, seven sets of primers were optimized and only one was suitable for the LAMP assay. To further confirm the results, the optimization test of LAMP primers was repeated twice and the results were consistent. Therefore, the primer set S2 was randomly selected for further tests in this study (Table S1, Fig. 1).

The appropriate concentration of LAMP reaction components was related to the parameters of LAMP primers. With the appropriate primer set S2, the concentration of LAMP reaction components was optimized and the appropriate results were obtained as shown in the section results. To optimize the reaction conditions of LAMP, we observed various temperatures from 57 °C to 68 °C. To detect the rapidity of the LAMP assay, the LAMP products were analyzed at intervals of 30 min. The results were visualized by color change and gel electrophoresis and showed that 60 °C to 64 °C was a suitable reaction temperature range and LAMP was amplified and visualized within 60 min. LAMP reaction could be accelerated and the reaction times would be shortened by the addition of loop forward (LF) and loop backward (LB) primers^15^. To improve the detection efficiency of LAMP, only LF primer was designed to accelerate the amplification reaction in this study. Our data showed that both the benzimidazole-resistant mutants and the wild type isolates of *S. sclerotiorum* were amplified when the additional LB primer was used in the LAMP assay (Data not shown). It is possible that the relatively high sequence identity of LB primer decreased the specificity of the LAMP reaction.

Resistance to benzimidazole fungicides has been reported in many phytopathogenic fungi and usually stems from specific point mutations in the *β*-tubulin gene^9^,^10^,^11^,^32^,^33^,^34^. *S. sclerotiorum* has only one point mutation genotype E198A, while *B. cinerea* has three point mutation genotypes E198A, E198K, and E198V. In *F. graminearum*, two homologous *β*-tubulins (*β*_*1*_ and *β*_2_) exist in the fungal genome and resistance to benzimidazole fungicides is due to a point mutation in the *β*_2_-tubulin gene, which has two point mutation genotypes E198K and E198L. To verify the specificity and efficiency of LAMP, these mutation genotypes were determined by the LAMP assay in this study. As shown in Fig. 5, the result was positive only for the E198A genotype of *F. graminearum*, and no positive sample of the LAMP assay was observed when these mutation genotypes were used as templates. Restriction enzyme and sequence analysis also validated its specificity (Fig. 2C). Meanwhile, the repeatability of LAMP was tested using 14 known E198A mutants (Table S2). All the samples were positive based on HNB-color change and gel electrophoresis (Fig. 7). Therefore, we concluded that the LAMP assay in this study had a good specificity and repeatability and was very suitable for detecting the benzimidazole-resistant mutants of *S. sclerotiorum* in field.

The sensitivity of LAMP was compared with PCR. The constructed plasmid pET383 was subjected to serial 10-fold dilutions to produce concentration ranging from 2 × 10^9^ copies to 2 × 10^0^ copies. As shown in Fig. 7, the detection limit of the LAMP assay was only 2 × 10^2^ copies, while that of PCR was 2 × 10^5^ copies, the developed LAMP method had 1000-fold higher sensitivity than that of the conventional PCR.

In this study, the primer set S2 has been used in the above described test. Other three LAMP primer sets (S3, S6, S8) were also determined for optimization of reaction temperature and time, specificity, sensitivity, repeatability and LB-acceleration tests. Surprisingly, the results from the primer sets (S3, S6, S8) were consistent with that from the primer set S2 (data not shown). Therefore, we concluded that all of the four primer sets from optimization test of LAMP primers could also specifically distinguish the benzimidazole-resistant isolates from the wild type isolates.

Traditional detection methods of the benzimidazole-resistant mutants of *S. sclerotiorum* have been reported in previous study^11^. To improve the detection efficiency and shorten the detection time, the LAMP assay was applied in monitoring benzimidazole-resistance populations of *S. sclerotiorum* in 2013. For comparison, the same samples were tested by PCR and MIC methods according to the previous studies^8^,^9^,^11^. In 2013, the resistance frequency by MIC, PCR and LAMP was 20.14%, 18.52% and 19.68%, respectively. Compared with PCR, the result by LAMP was more closed to that of MIC (Table 1). The detection of benzimidazole-resistant mutants of *S. sclerotiorum* by MIC is always time-consuming, which requires 2 to 5 days. Although PCR assay can shorten the detection time to 4 to 5 hours, it requires special and expensive equipment such as PCR thermocycler, electrophoresis set, and gel documentation system. In addition, the operation of PCR assay also requires researchers exposure to toxic materials such as ethidium bromide, gel-red and ultraviolet. However, the LAMP assay shortens the detection time to 1 hour and only requires a heating block, which is easily available in most laboratories. Furthermore, the result or product from the LAMP assay could be observed by naked eyes without requiring special and expensive equipments. Therefore, the established LAMP assay is simpler, more rapid and direct.

In the test of field samples in 2014, 629 positive samples were detected by the traditional MIC assay, and 614 positive samples by the LAMP assay. The resistance frequency by two methods was very similar (Table 2). In short, LAMP with HNB was shown to be rapid and easy for detection of the benzimidazole-resistant mutants of *S. sclerotiorum*. The LAMP assay will be a useful and promising technique for monitoring benzimidazole-resistant populations of *S. sclerotiorum* in the future. It can also play an important role in the early warning of resistance risk and resistance management of *S. sclerotiorum* in the field.

In brief, The LAMP assay combined with HNB was established and demonstrated to be more sensitive, specific, and practical for detection of the benzimidazole-resistant mutants of *S. sclerotiorum* than previous methods. Therefore, it will be potentially useful for monitoring the benzimidazole-resistant populations of *S. sclerotiorum* not only in the laboratory but also in the field.

## Methods

### Fungal isolates, culture conditions and reagents

*Sclerotinia sclerotiorum* isolate TZ25, which is highly resistant to MBC because of a point mutation at codon 198 (Glu to Ala, E198A) in the *β*-tubulin gene (GenBank: AY312374.1), *S. sclerotiorum* isolate JY4016, which is moderately resistant to MBC because of a point mutation at codon 200 (Phe to Tyr, F200Y) in the *β*-tubulin gene, and *S. sclerotiorum* isolate HA61, which is sensitive to MBC, were used in this study (Table S3). Information of the different resistant-level mutants of *Fusarium graminearum* and *Botrytis cinerea* to MBC is listed in the Table S3. All the *F. graminearum* and *B. cinerea* isolates were isolated by a single-spore method and all the *S. sclerotiorum* isolates were isolated by a single-sclerotia method.

PDA medium was used to culture *S. sclerotiorum* and in routine assays for sensitivity to MBC *in vitro*. Based on differences in MBC sensitivity, the tested isolates were divided into three phenotypes according to the minimum inhibitory concentration (MIC) method as follows: high MBC resistance, MIC > 100 μg mL^−1^; moderate MBC resistance, 100 μg mL^−1^ > MIC > 5 μg mL^−1^; MBC sensitive, MIC < 5 μg mL^−1^.

Carbendazim was provided by the Shenyang Academy of Chemistry and Industry, China. Bst DNA polymerase was purchased from NEB. Betaine and hydroxynaphthol blue (HNB) were purchased from Sigma, and MgCl_2_ and dNTPs were purchased from Takara. DNA marker (2K plus) was purchased from Transgen. Double-distilled water (ddH_2_O) was used in all experiments. All other reagents were analytical grade.

### DNA extraction

Genomic DNA of *S. sclerotiorum* was extracted from sclerotia and that of *F. graminearum* and *B. cinerea* was extracted from mycelia using the tissue lyser (MM400, Retsh) according to the CTAB procedure^35^,^36^.

### Primer design

PCR primers and LAMP primers were designed using Primer Premier 5.0 (Premier, Canada) and Primer explorer V4 software program (http://primerexplorer.jp/e/) according to previous studies^20^,^21^,^26^. Information of the primers used in this study is listed in the Table S1 and labeled in Fig. S1.

### Specificity of LAMP primers

Based on the single-point mutation at codon 198 (Glu to Ala, E198A) of the *β-*tubulin gene, a set of LAMP primers was designed using the Primer explorer V4 software program. To improve the specificity of LAMP amplification, basically a modified method was followed in which an artificial base pair mismatch was introduced within three nucleotides of the 5′ end of the forward inner primer (FIP) (Table S1). In this study, eight sets of LAMP primers (Table S1) were optimized to specially distinguish the E198A mutants from the wild types. With the genomic DNA of TZ25 and HA61 as a template, LAMP was performed at 63 °C for 60 min and then heated at 80 °C for 10 min to terminate the reaction. The positive reaction could be visually inspected based on HNB-visualized color change. In addition, LAMP product was also analyzed by agarose gel electrophoresis. Each treatment had two replications, and the experiment was repeated twice.

### Optimization of LAMP reaction components

To distinguish the E198A mutants from the wild types on basis of HNB-visualized color change, the concentration of LAMP reaction components was optimized in a total volume of 10 μL according to the previous described method^20^,^21^,^26^. As before, the LAMP assays were assessed based on HNB-visualized color change and gel electrophoresis. The test was repeated with three replicates for each treatment.

### Optimization of LAMP reaction conditions

According to the optimal LAMP reaction components, the LAMP reaction mixtures were incubated at 57, 58, 59, 60, 62, 63, 64, 66, 67 or 68 °C to determine the optimal reaction temperature. Then, the LAMP was performed at the desired reaction temperature for 15, 30, 45, 60, 75, 90, 105, or 120 min to determine the optimal reaction time. When the reaction was finished, the LAMP was assessed based on HNB-visualized color change and gel electrophoresis as described in the previous section. The test was performed three times with two replicates.

### Restriction enzyme digestion and sequence analysis of the LAMP products

To confirm that the LAMP products was the target fragment, LAMP products were analyzed by restriction enzyme digestion and sequencing. LAMP products was digested with the restriction enzyme Alu I (Takara) according to the operating instruction. The digested LAMP products were analyzed on agarose gel electrophoresis. Meanwhile, the 218 bp fragment was amplified by PCR using the primers F3 and B3 and cloned into pEASY^®^-T1 to create pET218 (Transgen, Beijing) for sequencing.

### Specificity of LAMP

Based on the optimal LAMP primer Set2, the specificity of LAMP was tested with genomic DNA of the wild type isolate HA61, the E198A mutant TZ25, the F200Y mutant JY4016 of *S. sclerotiorum*, the E198K mutant and the E198L mutant of *F. graminearum*, the E198V mutant, the E198A mutant, and the E198K mutant of *B. cinerea* (Table S3). The LAMP assay was performed and assessed as described in the previous section. The experiment was repeated twice with two replicates.

### Sensitivity of LAMP and PCR

Using the primers Ssbeta383F and Ssbeta383R, PCR was performed to amplify a 383-bp DNA fragment containing the point-mutation at codon 198 of the *β-*tubulin gene. The PCR products were isolated and cloned into pEASY^®^-T1 to create pET383 as above described method. The recombinant plasmid pET383 was extracted from positive clones and tenfold serially diluted 2 × 10^9^ copies to 2 × 10^0^ copies prior to its use as a template in LAMP and PCR. Primers Ssbeta383F and Ssbeta383R were used for PCR detection. The process and condition of the sensitivity of LAMP were similar to the specificity of LAMP described as above. When the reactions were completed, the products were analyzed on 3% agarose gel. The test was performed three times with three replicates.

### Repeatability of LAMP

According to the above described method, the accuracy of LAMP was performed with genomic DNA of 14 known *S. sclerotiorum* E198A mutants from different geographical regions in China (Table S2). The wild type HA61 and ddH_2_O were used as negative control under the same condition. The test was repeated twice.

### Comparison of LAMP, PCR and MIC

To evaluate the feasibility of the established LAMP method, comparison of different methods for detecting the E198A mutants of *S. sclerotiorum* was tested using 864 isolates from diseased rape stems from different fields of Jiangsu province of China in 2013 (Table 1). LAMP and MIC were performed as above described and PCR was performed following the method described by Li *et al.*^10^. To confirm that the positive samples had a point mutation at codon 198, PCR was performed to amplify the partial *β-*tubulin gene using the primers Ssbeta383F and Ssbeta383R. The PCR product was cloned into *pEASY*^®^-T1 and sequenced.

### Application of LAMP on monitoring MBC-resistance of *S. sclerotiorum* in field

To directly demonstrate the application of LAMP on resistance monitoring of *S. sclerotiorum* to MBC in agricultural production, a total of 1491 sclerotia were collected from diseased rape stems from different fields in Jiangsu province of China in 2014 (Table 2). These sclerotia were divided into two parts. One part was tested by LAMP and the other part was tested by MIC.

## Additional Information

**How to cite this article**: Duan, Y. *et al.* Development and application of loop-mediated isothermal amplification for detecting the highly benzimidazole-resistant isolates in *Sclerotinia sclerotiorum*. *Sci. Rep.* **5**, 17278; doi: 10.1038/srep17278 (2015).

## Supplementary Material

Supplementary Information

## Figures and Tables

**Table 1:** Comparation of LAMP, PCR, and MIC for detecting the highly MBC-resistant populations of *S. sclerotiorum* from different fields of Jiangsu province of China in 2013.

Geographical origin	Number of samples	LAMP		PCR		MIC	
		Positive	Resistance frequency (%)	Positive	Resistance frequency (%)	Positive	Resistance frequency (%)
Taizhou	387	76	19.64	72	18.60	78	20.16
Yancheng	117	23	19.66	24	20.51	23	19.66
Yangzhou	103	14	13.59	12	11.65	15	14.56
Suzhou	89	17	19.10	15	16.85	18	20.22
Huaian	104	25	24.04	24	23.08	25	24.04
Zhenjiang	64	15	23.44	13	20.31	15	23.44
Total	864	170	19.68	160	18.52	174	20.14

**Table 2:** Detection of the highly MBC-resistance populations of *S. sclerotiorum* by LAMP and MIC in 2014.

Geographical origin	Number of samples	Positive in LAMP	Resistance frequency by LAMP (%)	Positive in MIC	Resistance frequency by MIC (%)
Xuyi, Huaian	106	12	11.32	12	11.32
Rudong, Nantong	80	73	91.25	76	95.00
Zhangjiagang, Suzhou	96	48	50.00	48	50.00
Jianhu, Yancheng	96	34	35.42	36	37.50
Yizheng, Yangzhou	86	15	17.44	14	16.28
Tongzhou, Nantong	176	24	13.64	24	13.64
Jiangyan, Taizhou	193	109	56.48	111	57.51
Baimahu, Huaian	163	56	34.36	58	35.58
Sheyang, Yancheng	75	29	38.67	32	42.67
Haian, Nantong	88	32	36.36	32	36.36
Jurong, Zhenjiang	43	17	39.53	17	39.53
Liyang, Changzhou	35	6	17.14	6	17.14
Gaoyou, Yangzhou	115	88	76.52	89	77.39
Dongtai, Yancheng	64	42	65.63	42	65.63
Tangshan, Nanjing	75	29	38.67	32	42.67
Total	1491	614	41.18	629	42.19
