# Supplementary material for: Development and application of loop-mediated isothermal amplification for detecting the highly benzimidazole-resistant isolates in Sclerotinia sclerotiorum
Source: Sci Rep. 2015 Nov 26;5:17278. doi: 10.1038/srep17278 (PMC4660316; doi:10.1038/srep17278)
Supplement: Supplementary Information [file srep17278-s1.pdf]

Development and application of loop-mediated isothermal amplification for detecting the highly benzimidazole-resistant isolates in *Sclerotinia sclerotiorum*

Yabing Duan, Ying Yang, Jianxin Wang, Congchao Liu, Lingling He & Mingguo Zhou
College of Plant Protection, State & Local Joint Engineering Research Center of Green Pesticide Invention and Application, Nanjing Agricultural University, Nanjing, 210095, China

Correspondence and requests for materials should be addressed to M. G. Z. (mgzhou@njau.edu.cn)

Figure legend

Fig. 1 **Schematic illustration of LAMP primers for detection of the E981A mutants in *S. sclerotiorum*.** (A) Nucleotide sequence alignment of the target region *β*-tubulin gene in wild-type isolate HA61 and highly MBC-resistant isolate TZ25. The sequences used for LAMP primers are indicated by bold lines. One point mutation in black frame (GAG→GCG) leads to a high level of MBC resistance. (B) Schematic representation of the LAMP primers used in this study. Construction of the inner primers FIP and BIP are shown. F1c and B1c are complementary to F1 and B1, respectively.

Figure S1

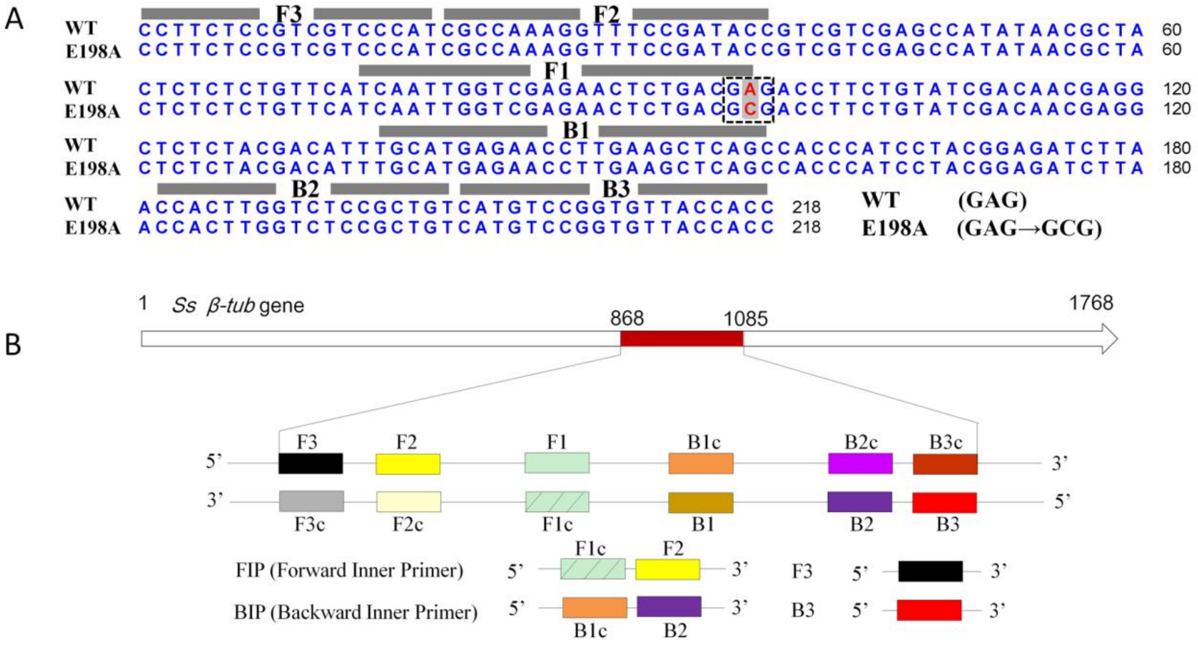

Supplementary Tables

Table S1. Information of the primers used in this study

Table S2. *Sclerotinia sclerotiorum* isolates used in repeatability test of LAMP

Table S3. Fungal isolates used in this study

Tables

Table S1

Primers	Sequence (5’-3’)[a]	Use
F3	CCTTCTCCGTCGTCCCAT	Forward outer primer for LAMP
B3	GGTGGTAACACCGGACATG	Backward outer primer for LAMP
BIP	TGCATGAGAACCTTGAAGCTCAGCACAGCGGAGACCAAGTGG	Backward inner primer for LAMP
LB	CACCCATCCTACGGAGATCTT	Loop backward primer for LAMP
FIP1	GCCGTCAGAGTTCTCGACCAATTGACGCCAAAGGTTTCCGATACC	
FIP2	G**G**GTCAGAGTTCTCGACCAATTGACGCCAAAGGTTTCCGATACC	
FIP3	GC**C**TCAGAGTTCTCGACCAATTGACGCCAAAGGTTTCCGATACC	
FIP4	GC**CA**CAGAGTTCTCGACCAATTGACGCCAAAGGTTTCCGATACC	Forward inner primers to distinguish the *S. sclerotiorum* genotypes (E198A) for LAMP
FIP5	**C**GCGTCAGAGTTCTCGACCAATTGACGCCAAAGGTTTCCGATACC	
FIP6	**TG**GCGTCAGAGTTCTCGACCAATTGACGCCAAAGGTTTCCGATACC	
FIP7	**TC**GCGTCAGAGTTCTCGACCAATTGACGCCAAAGGTTTCCGATACC	
FIP8	GG**G**GTCAGAGTTCTCGACCAATTGACGCCAAAGGTTTCCGATACC	
Ssbeta383F	TGATGTCGTTCGTCGTGAGGC	To amplify the partial fragments (383 bp) of the β-tubulin gene containing the E198A position.
Ssbeta383R	GACCAGGGAAACGGAGACAGG	

[a] Nucleotides in frames are modified from the sequence of the β-tubulin gene in the sensitive biotype and the resistant biotype. Nucleotides in bold are mismatches manually added specifically to distinguish *S. sclerotiorum* genotypes (E198A).

Table S2

Isolates	Origin	Host	Mutation of β tubulin	LAMP	MIC
XY4048	Jiangsu province, China	Rape	GAG→GCG, Glu to Ala, E198A	+	+
LA2015	Anhui province, China	Rape	GAG→GCG, Glu to Ala, E198A	+	+
HLJ1023	Heilongjiang province, China	Soybean	GAG→GCG, Glu to Ala, E198A	+	+
JZ2067	Hubei province, China	Rape	GAG→GCG, Glu to Ala, E198A	+	+
NT4082	Jiangsu province, China	Rape	GAG→GCG, Glu to Ala, E198A	+	+
QQ2351	Neimenggu, China	Sunflower	GAG→GCG, Glu to Ala, E198A	+	+
YZ24	Jiangsu province, China	Rape	GAG→GCG, Glu to Ala, E198A	+	+
YZ60	Jiangsu province, China	Rape	GAG→GCG, Glu to Ala, E198A	+	+
DQ2001	Heilongjiang province, China	Soybean	GAG→GCG, Glu to Ala, E198A	+	+
ZG4014	Jiangsu province, China	Rape	GAG→GCG, Glu to Ala, E198A	+	+
NT60	Jiangsu province, China	Rape	GAG→GCG, Glu to Ala, E198A	+	+
MAS238	Anhui province, China	Rape	GAG→GCG, Glu to Ala, E198A	+	+
CZ44	Jiangsu province, China	Rape	GAG→GCG, Glu to Ala, E198A	+	+
JH4008	Jiangsu province, China	Rape	GAG→GCG, Glu to Ala, E198A	+	+

Table S3

Fungal species	Isolates	Genotype description	Origin	Resistance phenotype [a]	LAMP [b]
S. sclerotiorum	HA61	Wild type	Jiangsu province, China	MBC^{S}	－
S. sclerotiorum	TZ25	Mutation at codon 198 of *β* tubulin (E198A)	Jiangsu province, China	MBC^{HR}	＋
S. sclerotiorum	JY4016	Mutation at codon 200 of *β* tubulin (F200Y)	Jiangsu province, China	MBC^{MR}	－
F. graminearum	J-2	Mutation at codon 198 of β_2 tubulin (E198K)	Anhui province, China	MBC^{HR}	－
F. graminearum	ZJ80	Mutation at codon 198 of β_2 tubulin (E198Q)	Anhui province, China	MBC^{HR}	－
B. cinerea	CGY004	Mutation at codon 198 of *β* tubulin (E198A)	Jiangsu province, China	MBC^{HR}	－
B. cinerea	SD2	Mutation at codon 198 of *β* tubulin (E198K)	Shandong province, China	MBC^{HR}	－
B. cinerea	SD4	Mutation at codon 198 of *β* tubulin (E198V)	Shandong province, China	MBC^{HR}	－

[a] MBC^{S}, MBC^{MR} and MBC^{HR} indicate that the isolate is sensitive, moderately and highly resistant to MBC, respectively.

[b] '＋' and '－' represent positive and negative results, respectively.
